# Cultivating new identities through horticulture, cooperation and mutual learning: the SOGNI project

**DOI:** 10.1192/j.eurpsy.2025.825

**Published:** 2025-08-26

**Authors:** A. Barbieri

**Affiliations:** 1 PSYCHIATRY, ASL CN1, CUNEO, Italy

## Abstract

**Introduction:**

Circular economy is a model of production and consumption entailing both environmental and social aspects. The collaborative dimension of this model is evident in the reuse of waste that can constitute a resource for other productive processes, encouraging cooperation and exchange. Also, the regeneration of disused spaces, in both urban and rural contexts, can stimulate social participation, cohesion and community development, thus becoming a resource for community-oriented interventions in the field of mental healthcare.

**Objectives:**

In 2024 a horticultural activity has been developed on the fields surrounding the facility, to grow edible species (mainly herbs and small fruits). The activity involved people hosted in the TC, local farmers, students of one agricultural high school and one catering school, to develop processes of agricultural products’ cultivation, exchange and transformation, as well as mutual learning. In particular, the project entails the reuse of TC’s organic waste (to produce compost), the cultivation of culinary plants and fruits, the production of derivatives (e.g. juices and jams) in the cooperation with the abovementioned schools, local producers, and consumers. The exchange of both products and skills aims to constitute (also metaphorically) fertile ground for the germination of new opportunities, supporting care in a broad sense and cultivating new individual and group identities. The original meaning of the acronym – “dreams” – points to the dreamlike dimension of a project that – within the controlled, safe perimeter of a pilot intervention – introduces innovative (sometimes bizarre) practices and unprecedented relationships between heterogeneous social actors, inviting the latter to “suspend every judgment”. Hopefully, configurations experienced during the pilot project and evidence supporting their therapeutic potential will be translated into the waking life, providing the basis for improved practice that intersects mental health, sustainability, and social cohesion.

**Methods:**

observational work and data collection conducted by healthcare professionals and an ethnographer.

**Results:**

The performance of practical tasks and the prolonged time of the activity throughout months provided each person with the time needed to gradually explore new relational spaces. The aim of growing food, a necessary step in this circular and collective enterprise, supported motivation as well as the development of a social identity of the group, who felt part of the wider community and actively involved in its development.

**Conclusions:**

Preliminary findings suggest further investigation and encourage collaboration between different social actors to develop inclusive, effective, and community-based interventions in the field of mental health and recovery.

**Disclosure of Interest:**

None Declared

